# Flavescence dorée phytoplasma infection represses the transcription of the lignin pathway in *Vitis vinifera* cv. Pinot noir without compromising monolignol accumulation

**DOI:** 10.3389/fpls.2026.1873582

**Published:** 2026-06-30

**Authors:** Christophe Debonneville, Aliona Hoste, Mathieu Mahillon, Frédéric Vuichard, Ágnes Dienes-Nagy, Isabelle Kellenberger, Jasmine Cadena i Canals, Olivier Schumpp

**Affiliations:** 1 Plant Protection, Agroscope, Nyon, Switzerland; 2 Wine Quality, Agroscope, Nyon, Switzerland

**Keywords:** flavescence dorée, lignification, metabolomics, shoots, transcriptomics, *Vitis vinifera*

## Abstract

Flavescence dorée phytoplasma (FDp) impairs shoot lignification in grapevines, but the underlying molecular mechanisms are unclear. This study examined how FDp infection affects the lignin/monolignol pathway in *Vitis vinifera* cv. Pinot Noir. Diseased (FDp positive) and healthy (FDp negative) shoots were sampled at three time points during the growing season. Metabolites were quantified by LC-MS/MS, key genes were analysed by RT-qPCR, and RNA sequencing was used to profile selected samples. FDp infection was found to reduce the expression of several lignin-biosynthetic genes and to broadly downregulate the phenylpropanoid pathway. Metabolomics revealed reduced levels of certain downstream intermediates, while shikimate and phenylalanine accumulated in infected shoots. However, the major monolignols coniferyl alcohol and sinapyl alcohol remained unchanged in mid- and late season. These results suggest that FDp represses transcription of the lignin pathway without substantially compromising monolignol accumulation, and that the lignification defect likely arises from downstream processes, such as precursor localisation or polymerisation.

## 1 Introduction

**Figure 1:**
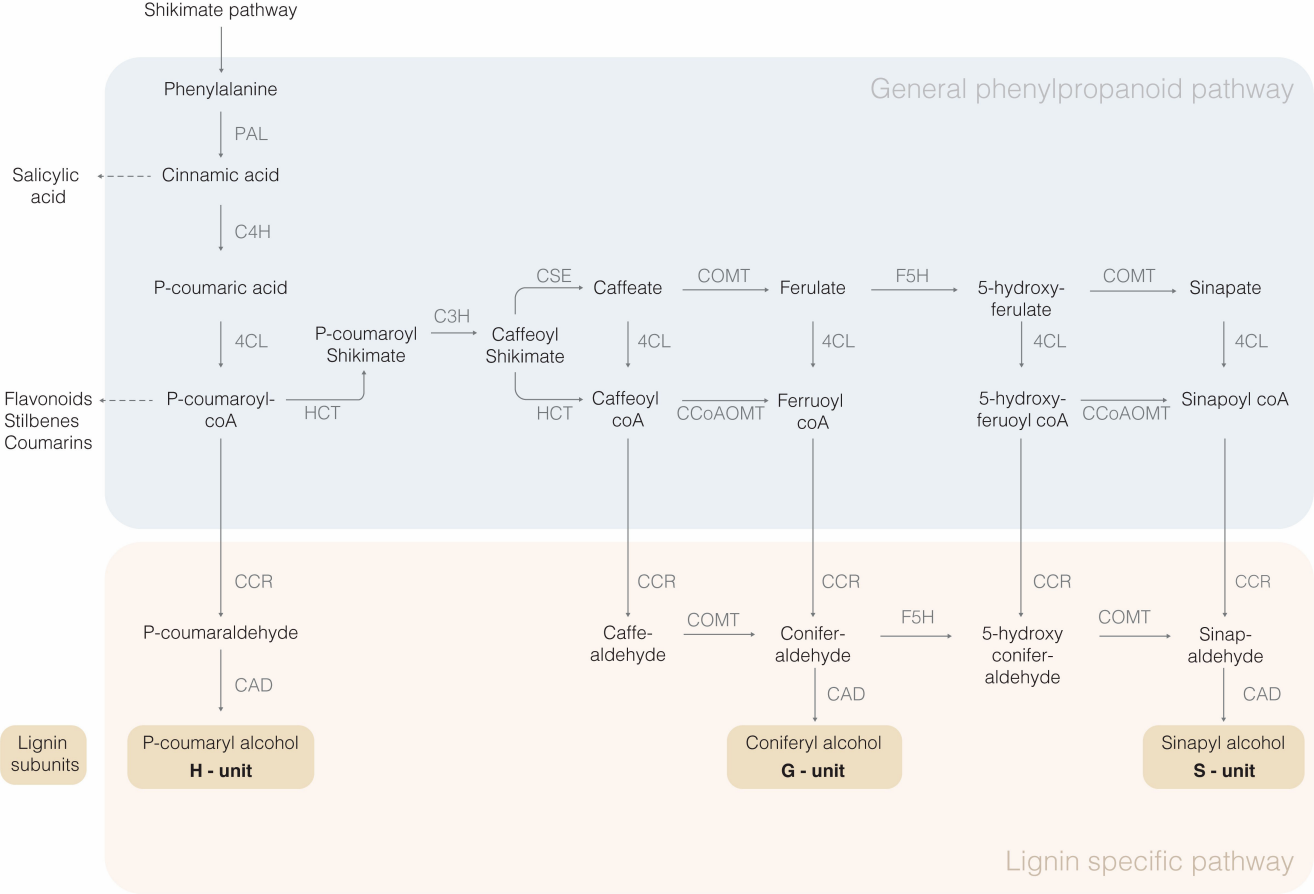
Schematic representation of the general phenylpropanoid and lignin-specific pathways. PAL, Phenylalanine ammonia-lyase; C4H, cinnamate 4-hydroxylase; 4CL, 4-coumarate:CoA ligase, HCT, shikimate Ohydroxycinnamoyltransferase; C3H, p-coumarate 3-hydroxylase; CSE, caffeoyl shikimate esterase; CCoAOMT, caffeoyl-CoA O-methyltransferase; COMT, caffeic acid 3-O-methyltransferase; ferulic acid 5-hydroxylase (F5H); CCR, cinnamoyl-CoA reductase; CAD, cinnamyl-alcohol dehydrogenase.

Phytoplasmas are wall-less prokaryotic pathogens closely related to gram-positive bacteria from the class Mollicutes (Doi et al., 1967). They cause numerous plant diseases worldwide (Bertaccini et al., 2014). As obligate intracellular parasites, they can only survive and multiply in the phloem of plants or the haemolymph of insect vectors, and they cannot be grown under axenic conditions. Phytoplasmas are mainly transmitted from plant to plant by sap-suckers from the order Hemiptera (Bertaccini et al., 2019). In grapevine (*Vitis* sp.), various phytoplasmas are associated with Grapevine Yellows (GY) (CABI, 2019). These diseases are present in all wine-growing areas, inducing significant economic losses. Flavescence dorée (FD) is one of the most harmful grapevine diseases (Tramontini et al., 2020) and is classified as a quarantine disease in the EU (EU, 2000) and Switzerland. It is associated with the Flavescence dorée phytoplasma (FDp), provisionally classified as ‘*Candidatus* Phytoplasma vitis’, and an epidemic propagation occurs only in the presence of the Nearctic leafhopper *Scaphoideus titanus* Ball (Chuche and Thiery, 2014). FD was first observed in France in the 1950s (Caudwell, 1957) and subsequently spread to other European countries. In Switzerland, the disease was first detected in the southern region Ticino in 2004 (Jermini et al., 2014) and expanded since then to other parts of the country. Despite the implementation of mandatory control measures, FD consistently spread in most of the affected countries. Typical symptoms of the disease are normally observed in late summer and consist of (i) downward rolling of the leaves with premature colour alterations (red and yellow for the red and white cultivars, respectively); (ii) inflorescence abortion or berry withering, and (iii) lack of lignification in new shoots. The lignification defect is characterized by shoots remaining green at the end of the season, which fail to rigidify, are flexible, and unable to withstand winter frost (Gullino, 2021). Phytoplasmas are known to induce profound developmental changes resulting in specific symptoms such as floral organs greening (virescence), transformation in leaf-like structures (virescence), and shoots proliferation (witches’ broom) (Hogenhout et al., 2008). Some of these symptoms are induced by secreted proteins, named effectors, released into host cells (Bai et al., 2009; MacLean et al., 2011; Sugawara et al., 2013; Sugio et al., 2011). These effectors manipulate phytoplasma’s insect and plant hosts. However, the recent release of the FDp genome has revealed the absence of coding sequences of known effectors (Debonneville et al., 2022). Some symptoms like downward leaf rolling with colour alterations, inflorescence abortion or berry withering are often associated with other pathogens (Wayne F. Wilcox et al., 2015), while lack of lignification, which is directly responsible for the decline of the canes, is unique to phytoplasma diseases. The presence of a pathogen usually has the opposite effect, contributing to increased lignin production to limit the development of bacterial or fungal phytopathogens (Lee et al., 2019; Mardi et al., 2015; Ninkuu et al., 2022; Zhang et al., 2025). The Stolbur phytoplasma is responsible for the symptoms of the ‘Syndrome Basses richesses’ disease in sugar beet, which results in increased lignin production (Gatineau et al., 2002), while FD-related phytoplasma strains cause hazel dieback without any visible alteration to lignification (Mehle et al., 2019). Lignin is one of the most abundant polymers found in vascular plants. Its deposition serves crucial physiological and developmental functions such as structural support and water transport in the vascular system. It is also essential for plant responses to biotic and abiotic stresses (Lourenço et al., 2016; Moura et al., 2010). Lignin is mostly made up of three monomers in various proportions, depending on the tissues, species and developmental stage, namely p-coumaryl alcohol (H-unit), coniferyl alcohol (G-unit), and sinapyl alcohol (S-unit). They are synthesized in the cytoplasm from aromatic acids of the shikimate pathway (i.e. phenylalanine) and transported to the cell wall where they are polymerized into lignin (Boerjan et al., 2003) (**Figure 1**). Although lignin production occurs in all plant species, the resulting lignin structures vary widely depending on the species, tissue, and stage of plant development (Dixon and Barros, 2019; Weng and Chapple, 2010). In grapevines, the proportion of the H subunit is very small both in fruits (Prozil et al., 2014) and shoots (Pereira et al., 2023). In other species such as gymnosperms, wood may consist of lignin without H subunits (Hiraide et al., 2021; Maeda et al., 2025). Lignin biosynthesis is regulated by a wide variety of transcription factors that activate or repress enzymes in this highly integrated metabolic pathway (Deng and Lu, 2017). In this study, a combined approach using metabolomics, untargeted transcriptomics, and gene expression analysis was used to identify the metabolic steps involved in the deregulation of the lignin biosynthesis pathway during FDp infection.

## 2 Materials and methods

### 2.1 Plant material

Vines were selected from a Pinot noir plot known to be affected by FD located in Roche, Switzerland. Three replicates of healthy (FDp negative) and diseased (FDppositive) shoots were collected. Healthy vines next to the infected ones were chosen. All vines were at the same development stage. Samples were collected at three different time points in 2022: on June 23^rd^, on September 1^st^, and on October 19^th^. The sampling was designed to span the seasonal development of FD symptoms and host responses: an early stage at the onset of visible symptoms, a mid-season stage when disease progression is established, and a late-season stage when symptoms are fully expressed (**Supplementary Figure 1**). Sections of about 20 cm of shoots were cut, immediately placed in liquid nitrogen, transported to the laboratory, and stored at -80 °C until processed. Their health status was confirmed using a quantitative PCR assay according to Pelletier et al., 2009 (Pelletier et al., 2009) (**Supplementary Table 1**).

### 2.2 Metabolomic analysis

The procedure of Jaini et al (Jaini et al., 2017). to quantify phenylpropanoid pathway intermediates in *Arabidopsis thaliana* served as a basis to develop the analytical method used in this work.

#### 2.2.1 Standard solutions and calibration curves

Of the 22 major compounds involved in the monolignol biosynthetic pathway, 13 were ordered according to their commercial availability. Shikimic acid (HY-N0130-100MG; 99.15%), L-phenylalanine (HY-N0215-200MG; 99.96%), trans-cinnamic acid (HY-N0610-50MG; 99.98%), p-coumaric acid (HY-N0351-100MG; 99.83%), ferulic acid (HY-N0060-100MG; 99.91%), sinapic acid (300977-20MG; ≥ 99%), coniferaldehyde (HY-N2535-5MG; 99.94%) and sinapaldehyde (HY-N1312-50MG; 99.96%) were obtained from Lucerna-Chem AG (Lucerne, Switzerland). Caffeic acid (205546-500; >98%), caffeoyl alcohol (PHL82489-10MG; >95%), p-coumaryl alcohol (PHL82506-10MG; >98%), coniferyl alcohol (41402-10MG; >97.5%), sinapyl alcohol (CAYM33916-50; >80%) and p-fluoro-dl-phenylalanine (F5251-1G; >98%), the internal standard (IS) for analyte loss corrections were supplied by Dr. Grogg Chemie AG (Stettlen, Switzerland). Analytes were prepared as a stock solution of 1 mg/ml in 100% methanol (MeOH). The 13 standards were then combined in a MeOH/H_2_O (75:25 v/v) solution each at a concentration of 5 μg/ml, except for phenylalanine (50 μg/ml), coniferyl alcohol (150 μg/ml) and sinapyl alcohol (250 μg/ml). The mixture was then diluted into 12 concentrations by serial dilution of 2. The IS was added to a concentration of 10 μg/ml in each dilution. Calibration curves of each metabolite were obtained by injecting 3 μl of the 12 dilutions into HPLC-MS/MS.

#### 2.2.2 Sample preparation and extraction

Sections of about 3 g from each sample were freeze-dried for 72 hours and ground to dust using a grinding machine. To protect samples from UV light and humidity, the powder was placed in individual amber glass bottles and stored in a container filled with silica gel. Each sample was extracted three times to obtain technical replicates. 50 mg of dried weight (DW) were transferred to a 1.5 ml Eppendorf tube and 1ml of 75% MeOH (MeOH/H_2_O 75:25 v/v) solution containing IS at a concentration of 10μg/ml was added (20μl of solution/mg of DW). The samples were immersed for five minutes at 45 kHz in an ultrasonic bath, vigorously vortexed, and incubated at 65 °C for 30 minutes. Each sample was then centrifuged for 15 minutes at 21,000 g, and 700 μl of the supernatant was transferred to a new Eppendorf tube, dried using an evaporator (SP Genevac EZ-2 Series), and re-dissolved in 100 μl of 75% MeOH solution.

#### 2.2.3 LC-MS/MS analysis

Samples and standards were separated by liquid chromatography (Agilent 1290 Infinity LC System) on a C18 column (150 mm x 4.6 mm, 2.7 μm, Agilent) using the following gradient of solvent A (0.1% formic acid water) and B (100% acetonitrile, 0.1% formic acid): 12% B (v/v) for 0–12 minutes, 12-25% B for 3 minutes, 25-100% B for 3 minutes, held 5 minutes at 100% B for washing and re-equilibrated to 12% B before subsequent injections. The injection volume was set at 3μl, the flow rate and column temperature were maintained at 1 ml/min and 30 °C respectively. Mass spectrometry with multiple reaction monitoring (MRM) was performed on an Agilent 6460 Series Triple Quadrupole operating in negative (ESI-) ionization mode. The source was configured with parameters used in the laboratory: 350 °C gas and sheath gas temperature, 13 l/min gas flow rate, 11 l/min sheath gas flow rate, 5000 V capillary voltage and 2000 V nozzle voltage. Collision energy and fragmentor voltage were optimized for each compound. Instrument control, data acquisition and data processing were performed using MassHunter 10.1 software (Agilent Technologie, Santa Clara, US). Quantification of analytes was carried out using *p*-fluoro-DL-phenylalanine as an internal standard. External calibration curves were obtained by analysing calibration mixture at 12 concentration levels. The preparation of the calibration mixture is described in Section 2.2.1. The linearity of each analyte was evaluated over the corresponding concentration range and was found to be satisfactory (R^2^ > 0.998). All data were analysed for statistical differences using GraphPad Prism 10 (version 10.4.1). Comparisons between groups were done using two-way ANOVA (multiple time-points), with Bonferroni *post-hoc* test for the effect of group. Technical replicates were averaged before statistical analysis.

### 2.3 RNA extraction

A total of 1–2 g of plant material was homogenized in 6 mL of extraction buffer (3% cetyltrimethylammonium bromide CTAB, 1.4 M NaCl, 25 mM EDTA, 100 mM Tris-HCl, 2% β-mercaptoethanol, pH 8.0). Subsequently, 2 mL of this homogenate were centrifuged for 10 min at 1000 x g. 900 μL of the supernatant were mixed with 2 μL of β-mercaptoethanol and shaken for 30 min at 600 rpm and at 65 °C. 900 μL of chloroform/isoamylalcohol (24:1 v/v)was added, homogenized by vortexing for 5 s and centrifuged for 5 min at 3000 x g. The aqueous layer was carefully transferred to a new tube, mixed with an equal volume of cold Isopropanol, and incubated 60 min at −20 °C for nucleic acid precipitation. Precipitated material was recovered by 2 min of centrifugation at 10,000 x g, 4 °C, and washed with 1 mL of cold 70% Ethanol. The pellets were dried for 2h at room temperature and resuspended into 100 μL of PCR-grade water. Samples were treated with DNAse I (RNase-Free DNase Set, Qiagen) following manufacturer’s instructions. RNA quality was assessed by capillary electrophoresis (Agilent 2100 bioanalyzer). Samples with an RNA Integrity Number (RIN) above 7 were selected for RT-qPCR analysis and Illumina sequencing.

### 2.4 Quantification of key enzymes from the monolignol pathway

#### 2.4.1 Gene selection and quantitative PCR primers and probes design

Enzymes from the phenylpropanoid/monolignol pathway were identified on KEGG (http://www.genome.jp/kegg/) and Phytozome (http://www.phytozome.net) databases. The following enzymes were chosen based on their position along the biosynthetic pathway and the small number of predicted isoenzymes: *V. vinifera* cinnamate 4-hydroxylase 1, 2, and 3 (*Vv*C4H-1, -2, and -3); *V. vinifera* p-coumarate 3-hydroxylase (*Vv*C3H); *V. vinifera* ferulic acid 5-hydroxylase 1, 2, and 3 (*Vv*F5H-1, -2, and -3); *V. vinifera* cinnamoyl-CoA reductase 1, 2, and 3 (*Vv*CCR-1, -2, and -3). Specific primer pairs and TaqMan probes were designed for each candidate using Geneious Prime v.2022.0.2 (**Supplementary Table 2**). When two isoenzymes’ sequences were too similar to design specific primers, consensus primers were designed (see VvC4H-2/VvC4H-3 and VvF5H-2/VvF5H-3). Each probe was modified with FAM fluorophore and BHQ-1 quencher.

#### 2.4.2 Reverse transcription- quantitative polymerase chain reaction

cDNA libraries were prepared using SuperScript IV First-Strand Synthesis System (Invitrogen) according to the manual instructions. Primer annealing was done with 500 ng RNA template, 1 μl 10 mM dNTP mix, 1μl 50 μM oligod(T) primer, and completed with H_2_O to a total volume of 14 μl. The mixture was heated at 65 °C for 5 min and incubated on ice while preparing the reverse transcription (RT) mix: 4 μl 5x SSIV buffer, 1 μl 100 mM DTT, 1 μl SuperScriptTM IV Reverse Transcriptase. Annealed RNA mixture was then combined to the RT reaction mix, incubated 10 min at 50 °C and 10 min at 80 °C for inactivation. First stranded cDNA library was created in a total volume of 20μl and was diluted eight times for a final volume of 160 μl. The simplex qPCR assays were carried out on CFX96 Touch System thermocycler (BioRad) and consisted of 7.5 μl 2x GoTaq Probe qPCR Master Mix (Promega), 4 μl H_2_O, 1.5 μl 10X primers/probe (200 nM primers and 100 nM probe final concentrations) and 1μl of cDNA template. Cycling was 5 min at 95 °C followed by 40 cycles of 15 sec at 95 °C and 30 sec at 60 °C. Each biological replicate was run in triplicates. Amplification efficiencies were determined by serial dilutions of the cDNA.

#### 2.4.3 Data analysis

Gene expression was normalised against two housekeeping genes, namely cytochrome oxidase (COX) and glyceraldehyde-3-phosphate dehydrogenase (GAPDH). These genes were previously identified as the two most stably expressed genes in healthy and FDp-infected grapevines by Bertazzon et al. (2019) (Bertazzon et al., 2019). Values are displayed as Normalized Expression (NE) determined using the following equation (Livak and Schmittgen, 2001):

Normalized Expression, (E_ref_)^Cq (ref)^/(E_target_)^Cq (target)^.

where E is the PCR efficiency, Cq is the cycle threshold, ref is the reference gene (either COX or GAPDH), and target is the target gene. All data were analysed for statistical differences using GraphPad Prism version 10 (version 10.4.1). Comparisons between groups were done using two-way ANOVA (multiple time-points), with Bonferroni *post-hoc* test for the effect of group. Technical replicates were averaged before statistical analysis.

Associations between RT-qPCR gene expression data (normalised against COX) and metabolite concentrations were assessed using Spearman correlation analyses. Correlations were calculated separately for healthy and diseased samples, with P-values adjusted using the Benjamini-Hochberg false discovery rate (FDR) procedure. Correlation matrices were generated to visualize gene-metabolite associations, and significant correlations (|ρ| ≥ 0.6, adjusted P-value < 0.05) were represented as network graphs. Correlation matrices and networks were performed in R 4 6.0.

### 2.5 Transcriptomics

RNA samples from September 2022 were sent to an external platform (https://www.macrogen-europe.com) for library preparation (TruSeq stranded mRNA kit) and sequencing (Novaseq 6000 platform; 150 bp paired-end reads). Raw reads were analysed with FastQC v0.11.9 (Andrews, 2010; FastQC: a quality control tool for high throughput sequence data. Available online at: http://www.bioinformatics.babraham.ac.uk/projects/fastqc) and poor-quality data, as well as adaptors, were removed with Fastp v0.23.2 (Chen et al., 2018). Reads assembly and mapping to the *Vitis vinifera* 12X.2 transcriptome (Canaguier et al., 2017) (**Supplementary Table 3**) and transcript quantification were performed with Salmon tool v1.9 (Patro et al., 2017) using default parameters. Differential gene expression analysis (log2 fold change) was performed with DESeq2 v1.37.6 (Love et al., 2014) on a public Galaxy server v22.05 (Galaxy, 2022) accessed in November 2024. Transcripts with False Discovery Rate (FDR) values lower than 0.05 and minimum fold change of 2 were considered as differentially expressed gene (DEG). Additionally, a principal component analysis (PCA) was conducted with DESeq2 to cluster samples according to their expression profile. The phenylpropanoid pathway was visualized using Pathview generated by Integrated Differential Expression & Pathway analysis (iDEP v2.01; (Ge et al., 2018)). A schematic representation of the workflow is presented in **Supplementary Figure 2** and read counts are provided in **Supplementary Table 4**. Illumina sequencing data have been deposited in the NCBI Sequence Read Archive (SRA) repository, PRJNA1458726.

## 3 Results

### 3.1 Analysis of metabolites from the monolignol pathway

**Figure 2:**
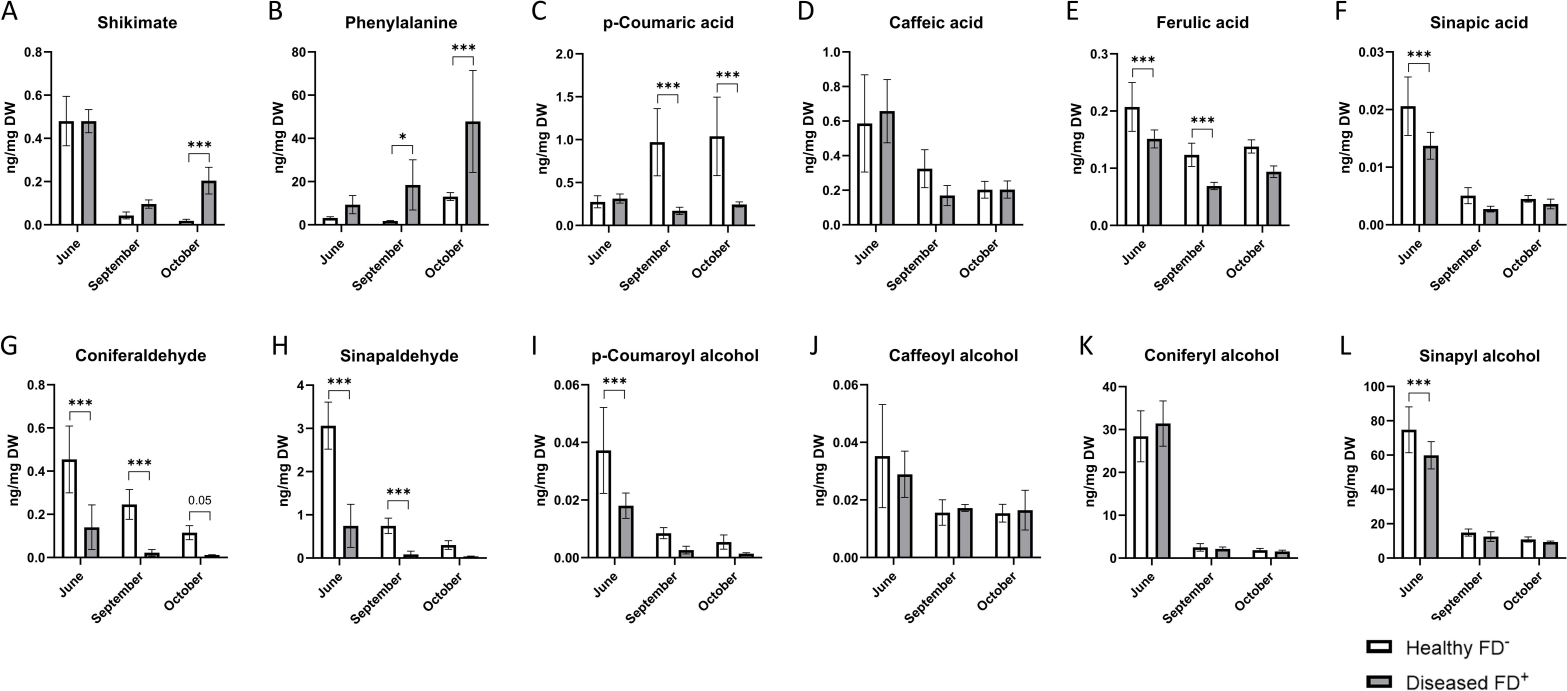
Concentration of metabolites **(A–L)** from the monolignol pathway at 3 time points through the vegetative season. Values expressed as ng/mg of dry weight (DW) are reported as mean ± SD. Comparisons between groups were done using two-way ANOVA (multiple time-points), with Bonferroni *post-hoc* test for the effect of group. (* p<0.05, *** p< 0.0001). H-unit=p-coumaryl alcohol, G-unit=coniferyl alcohol, and S-unit=sinapyl alcohol.

Thirteen intermediates of the phenylpropanoid/monolignol pathway were selected for investigation based on the commercial availability of pure compound. Only 12 were retained for analysis because levels of cinnamic acid in the samples were below the limit of detection in our experimental conditions. Several compounds, such as phenylalanine, coniferyl alcohol (subunit G) and sinapyl alcohol (subunit S), were present in significant quantities, in the order of several tens of ng/mg DW, while other compounds, such as coumaric alcohol (subunit H), sinapic acid, and caffeic acid, were present at concentrations in the pg/mg DW range. Nine compounds showed concentration differences between healthy and diseased shoots (p-value<0.05). Levels of two compounds, shikimate and phenylalanine, increased in infected shoots, with marked differences observed in the late season (October, **Figures 2A, B**). The concentration of shikimate was high early in the season, regardless of the studied condition, and decreased significantly as the season progressed. The process appeared slowed down in diseased shoots. In contrast, phenylalanine levels increased steadily throughout the season, with a notably greater accumulation in infected plants, reaching concentrations up to ten times higher than those observed in healthy plants. For several downstream metabolites, infection by FDp led to a significant decrease that could be observed as early as June. This was especially evident for ferulic acid, sinapic acid, coniferaldehyde, sinapaldehyde, p-coumaryl alcohol, and sinapyl alcohol (**Figures 2E–I, L** respectively). For some compounds, the decline in concentration in infected plants became increasingly pronounced over the course of the season, with coniferaldehyde and sinapaldehyde being almost undetectable in September and October. Levels of p-coumaric acid remained stable in infected plants throughout the season, whereas in healthy plants, concentrations increased fourfold in September and October compared to levels detected in June (**Figure 2C**). Caffeic acid, caffeoyl alcohol, and coniferyl alcohol did not exhibit any difference between healthy and diseased shoots during the entire vegetative season (**Figures 2D, J, K** respectively). Most of the compounds showed significant seasonal variations.

### 3.2 Quantification of the expression of key enzymes in the monolignol pathway

**Figure 3:**
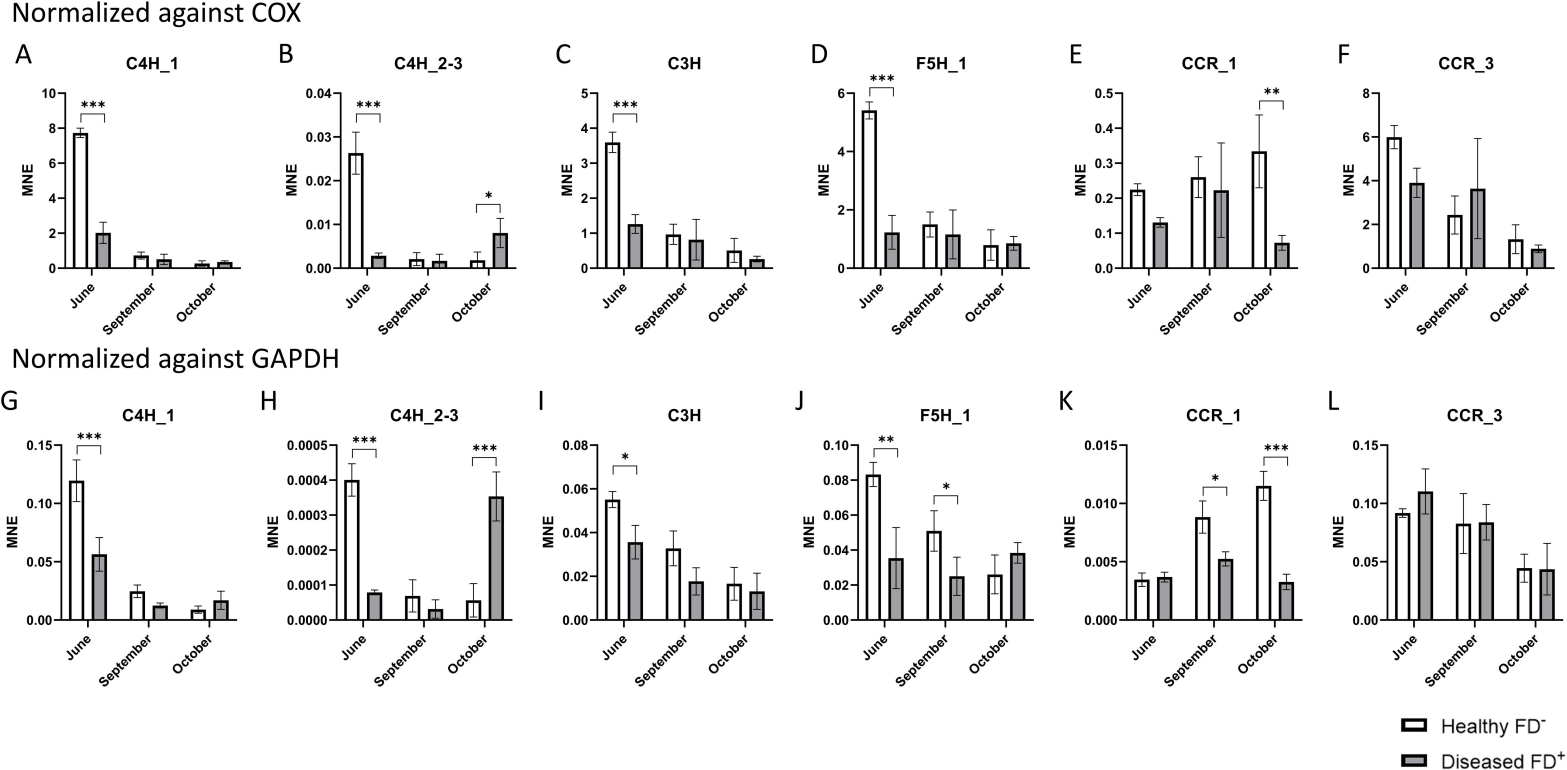
Mean normalized expression (MNE) of candidate genes from the monolignol pathway. Values normalised to the housekeeping genes COX **(A–F)** or GAPDH **(G–L)** are reported as mean ± SD. Comparisons between groups were done using two-way ANOVA (multiple time-points), with Bonferroni *post-hoc* test for the effect of group. (*p<0.05, **p< 0.01, ***p< 0.0001).

In parallel to the metabolic analysis, RT-qPCR was used to quantify the expression of genes involved in the monolignol pathway and to identify changes in their expression under FDp infection at the same time points throughout the vegetative season. In June, the expression of p-coumarate 3-hydroxylase (C3H), cinnamate 4-hydroxylase (C4H-1, C4H-2-3) and ferulic acid 5-hydroxylase (F5H-1) was significantly reduced in diseased shoots compared to healthy ones whether normalized with COX or GAPDH (**Figures 3A–D, G–J** respectively). On the other hand, the expression of both isoenzymes of cinnamoyl-CoA reductase CCR-1 and CCR-3 did not show significant differences in early season (**Figures 3E, F, K, L** respectively). However, CCR-1 showed reduced levels in late season (October) in infected samples whereas CCR-3 did not vary substantially between diseased and healthy vines. Isoenzymes 2 and 3 of cinnamate 4-hydroxylase (C4H-2-3), exhibited higher expression in infected samples in October (**Figures 3B, H**). However, the analysis cannot discriminate between the two isoenzymes. Most of the genes also showed significant seasonal variations. Expression of cinnamoyl-CoA reductase-2 and ferulic acid 5-hydroxylase-2 and 3 could not be detected in any of the conditions.

### 3.3 Correlation analysis of metabolites and RT-qPCR results

**Figure 4:**
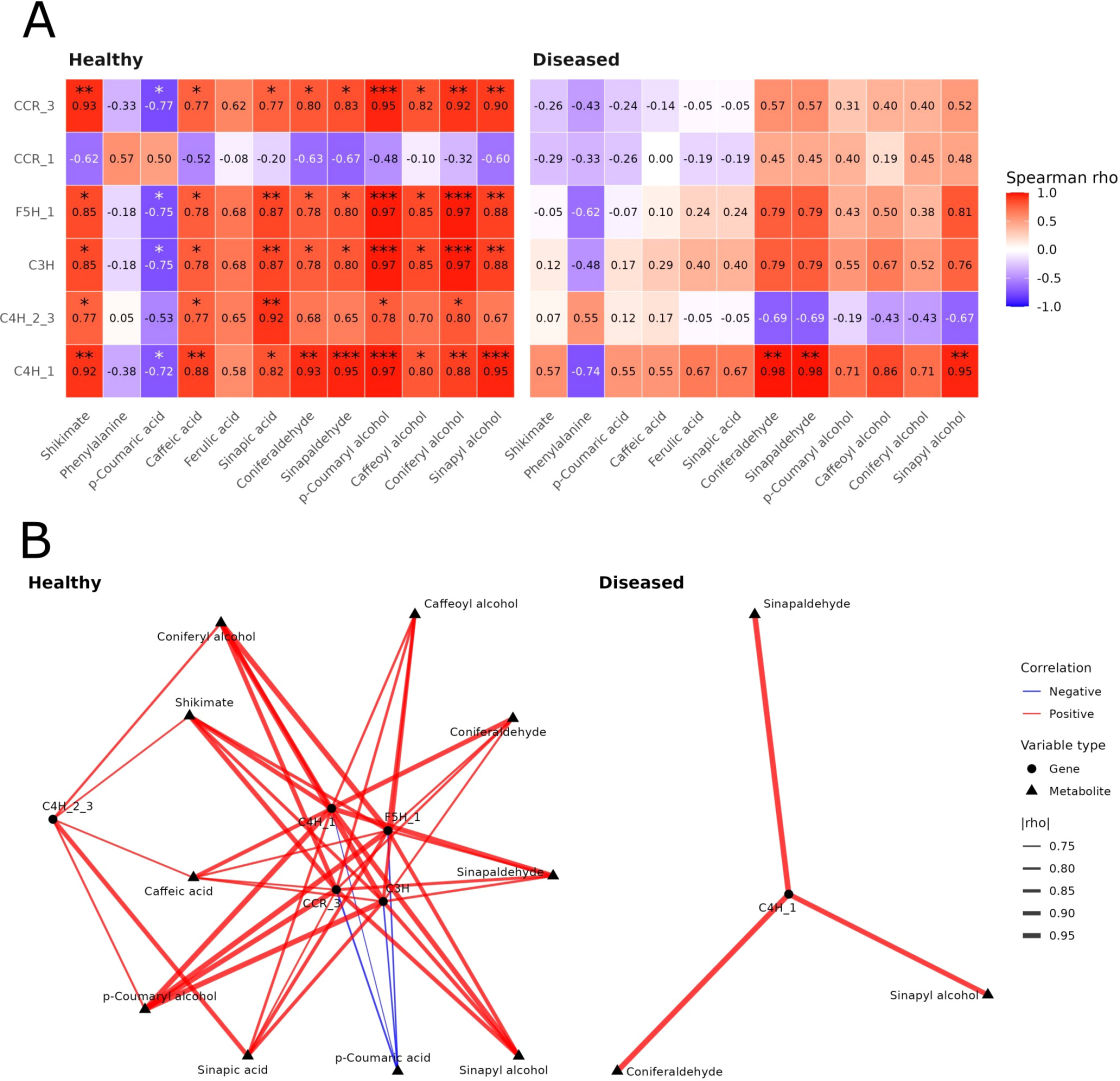
Spearman correlation analyses between lignin pathway genes and metabolites in healthy and diseased grapevines. **(A)** Correlation matrices. Values correspond to Spearman correlation coefficients (ρ), with blue indicating negative correlations and red indicating positive correlations. Asterisks denote significant correlations after Benjamini–Hochberg FDR correction (* adjusted P < 0.05, ** adjusted P < 0.01, *** adjusted P < 0.001). **(B)** Correlation networks. Only significant correlations (BH-FDR adjusted P < 0.05) are shown. Edge width is proportional to the absolute correlation coefficient (|ρ|), whereas edge colour indicates the direction of the correlation (blue, negative, red, positive).

The Spearman correlation analyses revealed marked differences between healthy and diseased plants (**Figure 4**). More significant correlations were detected in the healthy individuals than in the diseased ones (45 vs 3), and all the significant correlations exhibited a rho value of at least 0.77. Most of the correlations in healthy individuals were positive (41 out of 45), and so were the three in the diseased individuals. In healthy plants, several enzymes, namely C4H-1, C3H, F5H-1 and CCR-3 exhibited strong positive correlations with different metabolites, while in the diseased network most gene-metabolite associations were lost, and only C4H-1 remained significantly correlated with sinapaldehyde, sinapyl alcohol and coniferaldehyde. Overall, FDp infection markedly disrupted the coordination between lignin biosynthetic gene expression and lignin metabolite accumulation.

### 3.4 Transcriptomics

**Figure 5:**
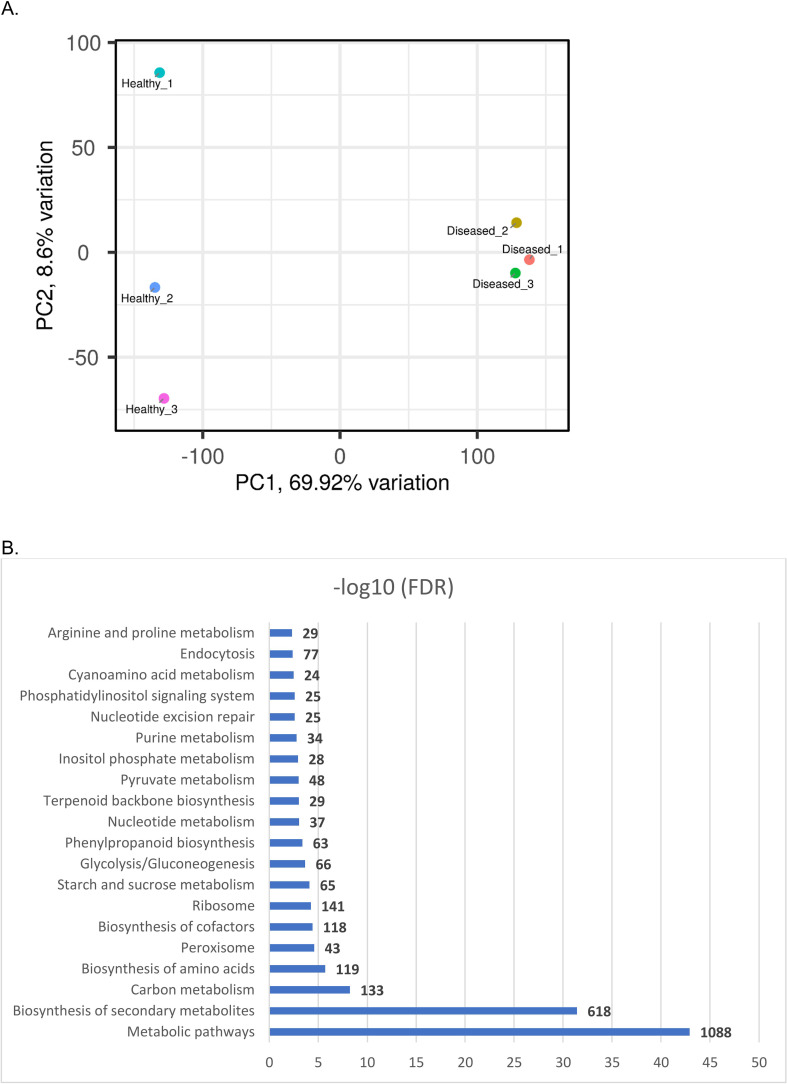
**(A)** Principal Component Analysis from 3 healthy (FDp negative) and 3 diseased grapevines. Samples collected in September were used. **(B)** Differentially expressed genes (DEG) enrichment analysis. The 20 most enriched KEGG terms are shown (false discovery rate FDR cut-off was set at 0.05). The number of genes involved are indicated besides the bar plots.

**Figure 6:**
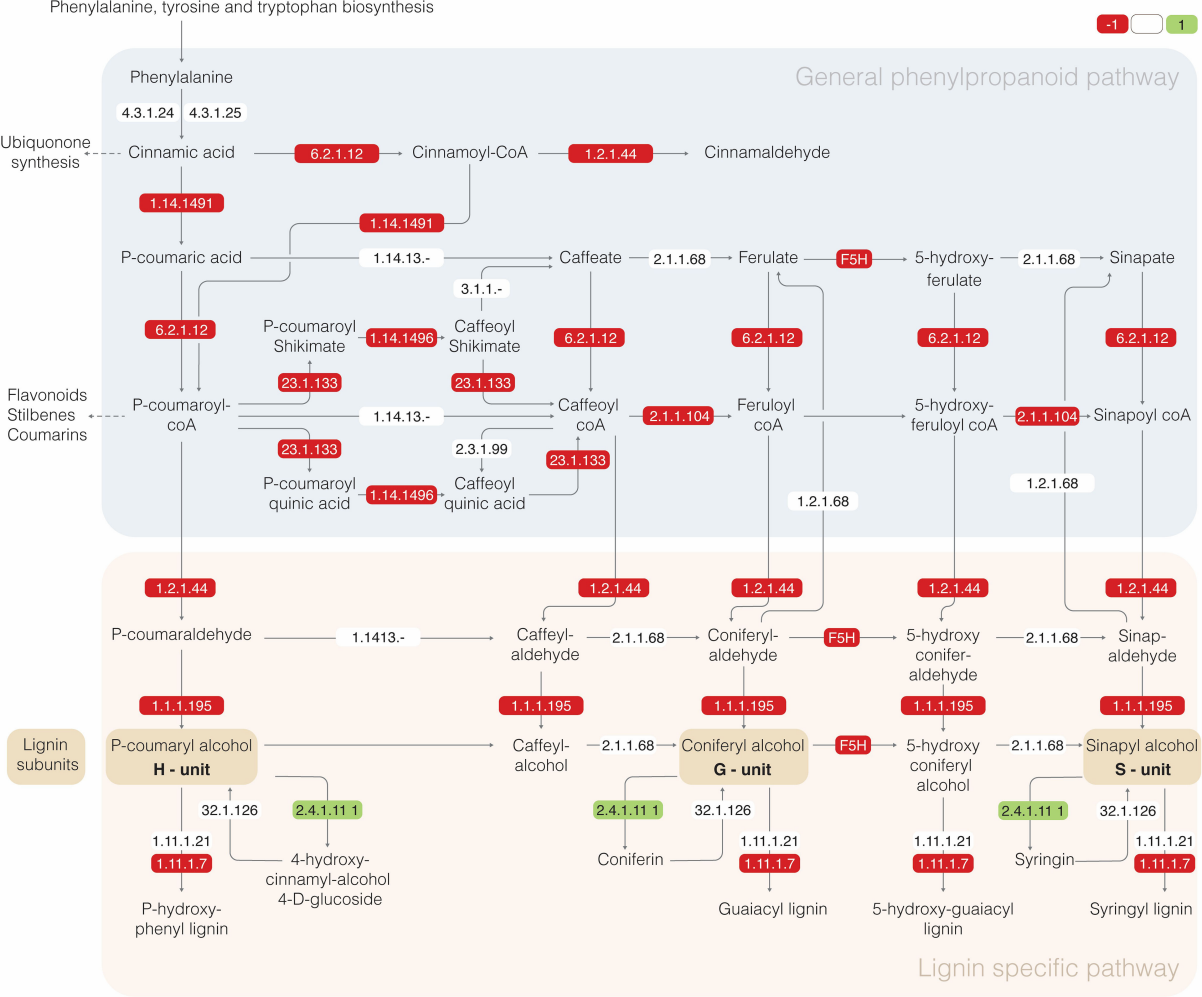
Fold change gene expression of the phenylpropanoid pathway visualised in KEGG graph using Pathview. All enzymes are indicated by framed numbers. Red square indicates downregulated genes and green square upregulated ones in diseased shoots vs. healthy. White square indicates unregulated or undetected genes. 1.14.1491, *Vv*C4H; 1.14.1496, *Vv*C3H, and 1.1.1.195, *Vv*CCR. **Supplementary Table 6** provides a full list of enzymes with their corresponding EC numbers.

RNAs extracted from three healthy and three diseased shoots harvested in September were used for shotgun transcriptomics by Illumina sequencing. Comparison of transcriptional profiles was performed with a principal component analysis (PCA). Principal Component 1 (PC1) explained 69.9% of the variance separating diseased and healthy shoots (**Figure 5A**), confirming differential gene expressions relative to the infectious status. Principal Component 2 (PC2) accounted for 8.6% of the variance in samples within a single condition. Out of more than 42,000 genes, 6,442 were differentially expressed (FDR < 0.05) in infected shoots compared to healthy ones. A total of 4,054 genes were upregulated, whereas downregulation was evidenced for 2,388 genes. Enrichment analysis of KEGG pathways revealed that “Metabolic pathways” was the most significantly affected category (**Figure 5B**). Different pathways associated with amino acids biosynthesis and carbon/carbohydrate metabolism as well as “Phenylpropanoid biosynthesis” were highlighted in the 20 most represented groups (**Supplementary Table 5**). A closer examination of the phenylpropanoid pathway in Pathview confirmed its overall downregulation (**Figure 6**). Moreover, **Table 1** shows log2 fold change of selected enzymes from the monolignol pathway as determined by DESeq2 analysis further evidencing the downregulation. RNA seq data also corroborated that *Vv*CCR-2 and *Vv*F5H-2/*Vv*F5H-3 were not expressed. No reads were detected. Some Myb transcription factors, which regulate secondary cell wall formation by influencing the biosynthesis of lignin and other compounds, were also differentially expressed (**Table 2**). Myb4, a repressor of lignin biosynthesis, is slightly, although not significantly, overexpressed in diseased plants. By contrast, the expressions of certain R2R3-Myb subfamily transcription factors such as Myb20, Myb43, Myb58, and Myb85, which are all activators of lignin biosynthesis, were strongly reduced in infected plants.

**Table 1:** log2 fold change (log2 FC) in the expression of selected enzymes from the phenylpropanoid pathway.

Enzyme	Gene IDs		log2 (FC)	P-value
*Vv*C3H	Vitvi08g00940	VIT_08s0040g00780	-2.1	8.53E-09*
*Vv*C4H_1	Vitvi06g00803	VIT_06s0004g08150	-1.57	2.40E-06*
*Vv*C4H_2	Vitvi11g01045	VIT_11s0078g00290	-3.57	1.57E-04*
*Vv*C4H_3	Vitvi11g00924	VIT_11s0065g00350	-0.88	0.38
*Vv*F5H_1	Vitvi04g01412	VIT_04s0023g02900	-0.01	0.99
*Vv*F5H_2	Vitvi03g00040	VIT_03s0038g00550	n/a	n/a
*Vv*F5H_3	Vitvi03g00038	VIT_03s0038g00500	n/a	n/a
*Vv*CCR_1	Vitvi06g00699	VIT_06s0004g07130	-1.84	5.40E-28*
*Vv*CCR_2	Vitvi12g00011	VIT_12s0142g00510	n/a	n/a
*Vv*CCR_3	Vitvi09g00993	VIT_09s0070g00240	-0.93	0.0012*

*A P-value of less than 0.05 is considered to indicate a significant fold change.

A negative log2 (FC) value means downregulation in diseased plants.

**Table 2:** log2 fold change (log 2 FC) in the expression of some Myb domain transcription factors.

Protein name	Gene IDs		log2 (FC)	P-value
Myb domain protein 4	Vitvi01g00401	VIT_01s0011g04760	0.68	0.112
Myb domain protein 4	Vitvi03g00136	VIT_03s0038g02310	0.24	0.611
Myb domain protein 4	Vitvi04g01486	VIT_04s0023g03710	1.65	0.279
Myb domain protein 3	Vitvi08g02196	VIT_08s0007g00360	-0.93	0.618
Myb domain protein 15	Vitvi05g01733	VIT_05s0049g01020	3.84	1.54E-05*
Myb domain protein 42/85	Vitvi02g01823	VIT_00s1241g00010	-3.91	4.10E-12*
Myb domain protein 58/63	Vitvi16g01449	VIT_16s0039g01920	-0.55	0.047*
Myb domain protein 58/63	Vitvi19g01669	VIT_19s0085g00050	-2.29	1.70E-05*
Myb domain protein 20/43	Vitvi14g02430	VIT_14s0006g00450	-1.48	2.07E-06*
Myb domain protein 94	Vitvi17g00598	VIT_17s0000g06190	-1.88	2.83E-04*
Myb domain protein 73	Vitvi07g01676	VIT_07s0129g01050	-0.25	0.249
Myb domain protein	Vitvi01g00867	VIT_01s0026g01050	-0.67	0.051
Myb domain protein	Vitvi17g00366	VIT_17s0000g04130	-1.32	4.35E-03*
Myb domain protein	Vitvi16g01215	VIT_16s0050g02530	-0.07	0.925

*A P-value of less than 0.05 is considered to indicate a significant fold change.

A negative log2 (FC) value means downregulation in diseased plants.

## 4 Discussion

Impaired lignification is a symptom unique to grapevine yellows associated with phytoplasmas. In this study, we focused on visible defects in the lignification process on vine shoots. The levels of metabolites and the expression of enzymes from the monolignol pathway were studied in healthy and diseased Pinot noir canes. Infection with FDp causes early and significant disruption of secondary metabolic pathways, observable through a decrease in the expression of several enzymes in the monolignol metabolic pathway as well as in the concentration of several intermediate metabolites (**Figures 2**, **3**). A correlation analysis highlighted a strong decoupling between gene expression and metabolite accumulation in diseased shoots (**Figure 4**). On the other hand, our data also revealed that shikimic acid and phenylalanine, located upstream of the phenylpropanoid pathway, had higher levels in infected shoots, especially in October. It has already been shown that the concentration of phenylalanine is influenced by bacterial and phytoplasma infections (Jan et al., 2020; Li et al., 2022). Our results suggest that processing of phenylalanine further in the pathway may be impaired in diseased shoots. Indeed, previous studies revealed that genes involved in phenylpropanoid biosynthesis are up-regulated in Bois noir phytoplasma-infected *Vitis* sp leaves (Albertazzi et al., 2009; Hren et al., 2009; Negro et al., 2020) favouring the synthesis of flavonols, stilbenoids, and certain phenolic compounds (Rusjan and Mikulic-Petkovsek, 2014). This strategy is generally used by plants as a defence mechanism against pathogen infection. As the synthesis of lignin and these compounds requires the same precursors (**Figure 1**), it is suggested that their excessive use in certain biosynthetic processes may reduce lignin production. Thus, the default of lignification observed in diseased canes would be a consequence of the plant response to phytoplasma infection. Moreover, several transcription factors involved downstream in the lignin biosynthesis pathway were repressed in infected plants. These studies suggested that impaired lignification of the young shoots could also result from this downregulation.

Nevertheless, our analyses show that the levels of the two main lignin subunits (G and S) in infected vines are comparable to those in healthy plants at mid- and late stages of the season, whereas the quantity of the H subunit decreases throughout the season (**Figure 2**). Intriguingly, several studies have shown that lignin synthesised in response to biotic or mechanical stresses, known as “stress lignin”, has the characteristic of higher amounts of H unit (Cesarino, 2019; Kim et al., 2020). This process is dependent on R2R3-MYB subfamily transcription factors known to be activators of lignin biosynthesis. Notably, Myb15 is specifically required for basal immunity and effector-triggered immunity involving lignification response upon bacterial challenge. Interestingly, our RNA-seq data from samples collected in September show that Myb15 is strongly upregulated in diseased plants, in contrast to other R2R3 transcription factors that activate lignin synthesis (Myb20, Myb43, Myb58, Myb85). On the other hand, Myb4, a repressor of lignin synthesis, is slightly upregulated in infected plants. This may also be a strategy used by FDp to counteract plant defence. Indeed, numerous pathogens, including fungi and bacteria, produce effectors influencing the expression of genes involved in the lignification process (Bauters et al., 2021). Further investigation is needed to elucidate the complex lignification processes in *Vitis vinifera*, and to determine whether a reduction in H lignin levels could result in the observed impaired lignification in infected vines.

Beside these specific responses, numerous studies have shown that the composition of lignin adapts to the availability of monolignols at the polymerization site (Blaschek et al., 2023; Fournand et al., 2003; Ishida et al., 2024; Tobimatsu and Schuetz, 2019). The comparable quantities of monolignols found in healthy and infected plants suggest that the defect in lignification may occur after the monolignol subunits are produced. These subunits, synthesised in the cytoplasm, are exported to the apoplast where they undergo polymerisation to form lignin in a reaction catalysed by either laccases or peroxidases. The transport of monolignols to the lignin polymerization site has long remained a mystery because it is technically difficult to study. Perkins et al (Perkins et al., 2022). demonstrated that monolignols diffuse along a gradient between the cytoplasmic production zone and the zone of utilisation by oxidative enzymes associated with the apoplastic cell wall in the lignified xylem of *Arabidopsis* roots. However, other recent work on spruce cell cultures used as a model for lignification suggests the existence of other mechanisms. In this model, monolignols are delivered to the apoplast via extracellular vesicles (Kankaanpaa et al., 2024). This model of active transport of lignin subunits has also been demonstrated in *Arabidopsis* leaves, in which autophagic vesicles are necessary for the synthesis of lignins involved in the defence response to infection by the bacterium *Pseudomonas syringae* DC3000 (Jeon and Segonzac, 2023). In parallel, work carried out on gymnosperm wood (Hiraide et al., 2021) and *Arabidopsis* stems (Hoffmann et al.; Tobimatsu and Schuetz, 2019; Yi Chou et al., 2018) show that the localisation of enzymes responsible for the formation of phenolic radical precursors by oxidation and the localized production of H_2_O_2_ are also decisive in controlling the lignification process. To identify the mechanisms by which GY phytoplasma inhibit the lignification process in grapevines, it is necessary to study the localisation of these precursors or enzymes. This is a complex task, as these enzymes are highly redundant with a very large number of isoforms, 17 for laccases and more than 73 for peroxidases in *Arabidopsis* (Rojas-Murcia et al., 2020). Nevertheless, the hypothesis of a blockage in the export of monolignols is also supported by the stimulation of UDP-glucose:coniferyl-alcohol 4’-beta-D-glucosyl production enzymes observed in transcriptomic analyses (**Figure 6**, enzyme 2.4.1.111). This storage of monolignols in glycolyzed form aims to limit their toxic accumulation in the cytoplasm of cells. The production of glucosyl-monolignols and their storage in the vacuole is a protective mechanism observed in cell cultures subjected to high concentrations of coniferyl alcohol and in plants genetically modified to accumulate high amounts of monolignols (Perkins et al., 2022; Vaisanen et al., 2015).

The numerous alterations observed at various stages of the phenylpropanoid pathway and upstream in the shikimate pathway, which lead to the biosynthesis of monolignols, do not appear to significantly impact the production of the primary lignin subunits, G and S. This apparent stability could be explained by the functional redundancy of the enzymes involved, which are often encoded by several homologous genes (Dixon and Barros, 2019). However, these metabolic pathways also contribute to the production of a wide range of secondary compounds, some of which are closely associated with interactions between plants and pathogens.

## 5 Conclusion

This study used multiple approaches to reveal that the lignin-specific pathway was downregulated in FD-diseased plants. This led to an unexpected accumulation of monolignol precursors, despite the typical reduction in lignin deposition in secondary cell walls. Our data suggest that disturbances to metabolism and transcription do not specifically target monolignol synthesis. This indicates that key determinants of the lignification phenotype may lie in processes such as the localisation of enzymes or precursors, rather than in enzyme abundance alone. Deeper comparative study between BNp and FDp effector repertoires could provide further insights into the underlying mechanisms given that these two pathogens cause the same symptoms. Furthermore, investigations on cultivars with variable susceptibilities to FDp will help to clarify which grapevine genetic characteristics impact phytoplasma multiplication, symptoms severity, and disease incidence, and provide hints on the genetic basis of FD susceptibility.

## Data Availability

The datasets presented in this study can be found in online repositories. The names of the repository/repositories and accession number(s) can be found below: https://www.ncbi.nlm.nih.gov/, PRJNA1458726.

## Supplementary material

Supplementary Figure 1 Illustration of Flavescence dorée symptoms on Pinot noir. **(A)** In June, the first symptoms appear on leaves; the shoots are still immature. **(B)** Plant showing FD symptoms, which can be seen next to healthy ones in September. **(C)** Cane from a diseased plant (immature, green) next to canes from a healthy one (lignified, brown) in October.

Supplementary Figure 2 Schematic representation of the workflow used for the analysis of RNA-seq (modified from http://bioinformatics.sdstate.edu/idep20/).
