# Supplementary figures and images for: Flavescence dorée phytoplasma infection represses the transcription of the lignin pathway in Vitis vinifera cv. Pinot noir without compromising monolignol accumulation
Source: Front Plant Sci. 2026 Jun 30;17:1873582. doi: 10.3389/fpls.2026.1873582 (PMC13364842; doi:10.3389/fpls.2026.1873582)

A.

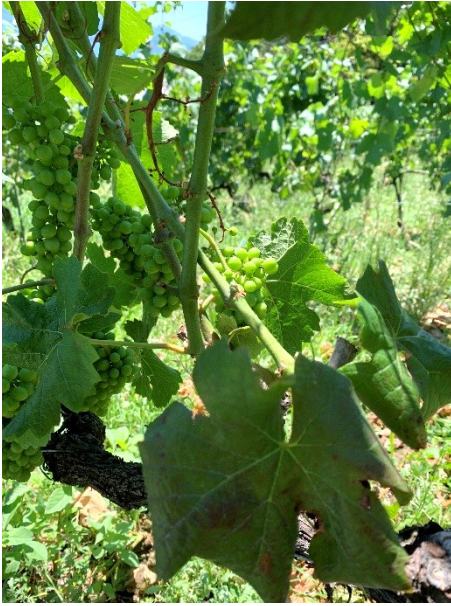

B.

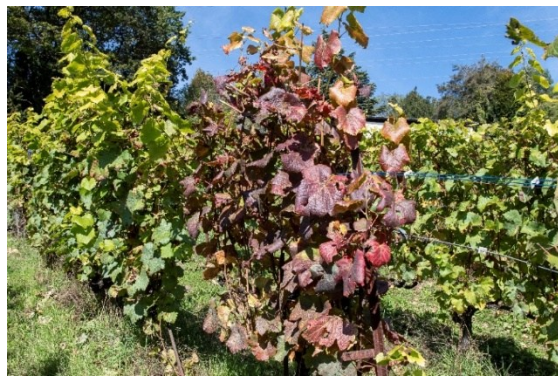

C.

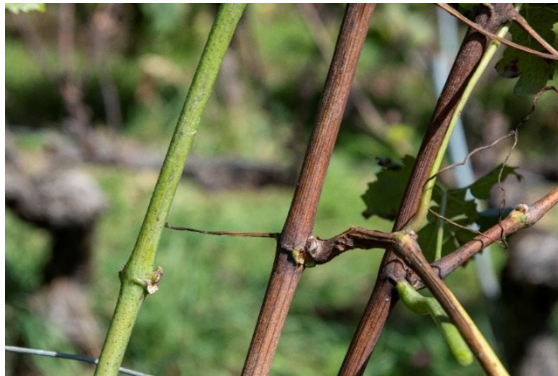

Supplement: Supplementary Figure 1 — Illustration of Flavescence dorée symptoms on Pinot noir. (A) In June, the first symptoms appear on leaves; the shoots are still immature. (B) Plant showing FD symptoms, which can be seen next to healthy ones in September. (C) Cane from a diseased plant (immature, green) next to canes from a healthy one (lignified, brown) in October. [file DataSheet1.pdf]

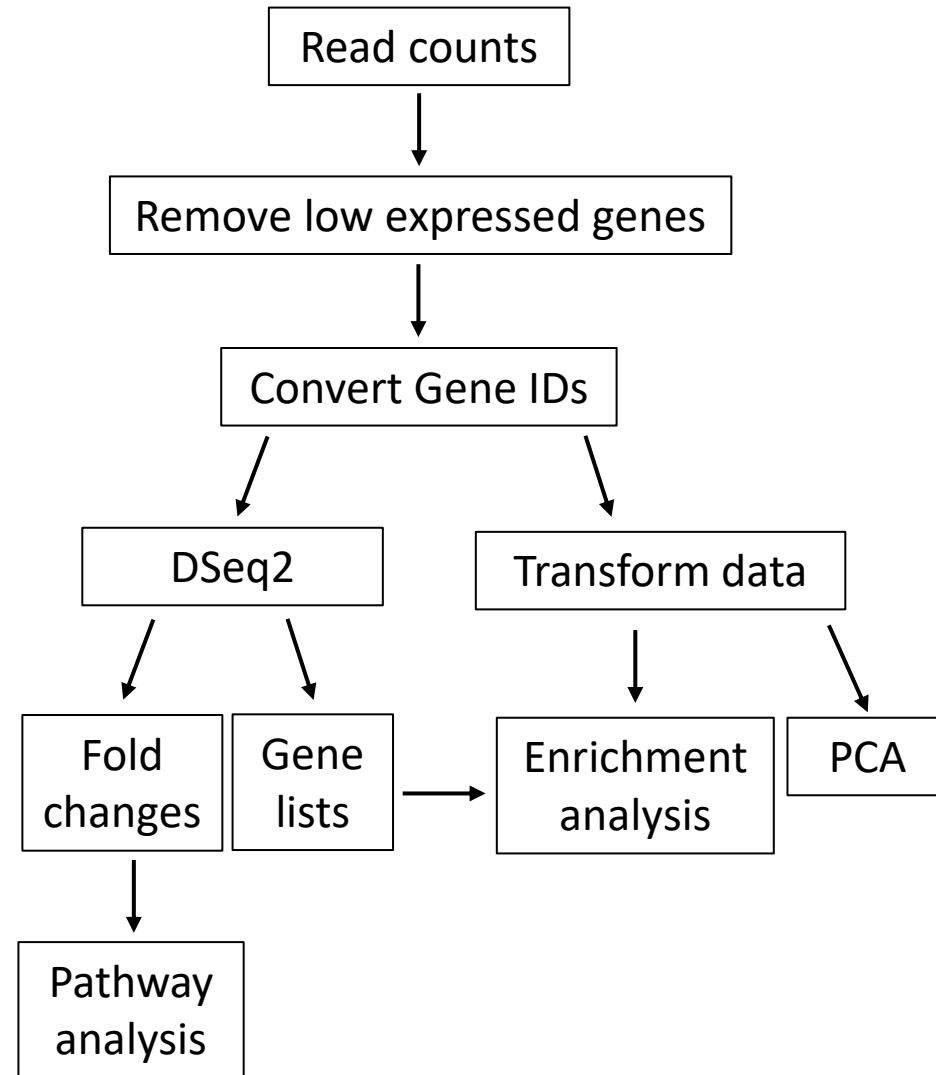

Supplement: Supplementary Figure 2 — Schematic representation of the workflow used for the analysis of RNA-seq (modified from http://bioinformatics.sdstate.edu/idep20/). [file DataSheet2.pdf]
